# Sexual orientation and gender identity data: An observational study assessing the feasibility of SOGI collection in clinical research and patient assistance programs

**DOI:** 10.1371/journal.pone.0332805

**Published:** 2025-10-22

**Authors:** Isabel Brown, Thuan Tran, Audrey Funwie, Shilpen Patel, Keith Dawson, Meghan McKenzie

**Affiliations:** 1 Genentech, Inc, South San Francisco, California, United States of America; 2 Department of Global Health, University of Washington, Seattle, Washington, United States of America; Virginia Commonwealth University, UNITED STATES OF AMERICA

## Abstract

**Purpose:**

Sexual and gender minority (SGM) people often experience significant health disparities and poor health outcomes. The objective of this study was to assess the feasibility and outcomes of sexual orientation and gender identity (SOGI) data collection in 2 distinct survey settings: (1) clinical research and (2) patient assistance programs.

**Materials and methods:**

In this survey study, participants completed 1 of 2 separate, distinct, self-reported, optional, United States–based surveys: (1) an in-person survey at clinical research sites for a cough or cardiac study between December 2022 to December 2023 (sponsored by Genentech) or (2) a web-based patient assistance program survey from a patient assistance program between January 2023 to December 2023 (sponsored by the Genentech Patient Foundation).

**Results:**

Of 33 clinical research survey participants, 100% of participants answered SOGI-related questions, leading to a non-response rate of 0%. Most (87.88%) participants identified as cisgender; 84.85% identified as heterosexual, 3.03% bisexual, 3.03% gay, 3.03% questioning, and 6.06% preferred not to answer.

Of 8499 patient assistance program survey participants, 99.54% answered gender identity questions (non-response rate of 0.46%) and 95.92% answered sexual orientation questions (non-response rate of 4.08%). Gender identities in the patient assistance program survey included 61.30% female, 34.22% male, 0.12% genderqueer, 0.06% transgender male, 0.14% none of the options, and 4.16% “Prefer not to answer.” Self-reported non-heterosexual orientations included bisexual, gay, lesbian, queer, questioning, “Prefer not to answer,” and something else.

**Conclusions:**

These findings suggest that patients in both survey settings are willing to self-disclose sexual orientation and gender identity information. These results underscore the rationale for adopting SOGI data collection in these settings.

**Clinical trials:**

NCT05660850, ISRCTN10520571

## Introduction

Sexual and gender minority (SGM) people often experience significant health disparities, including lower life expectancy, increased incidence of disease, and higher rates of mental health conditions [1–5]. Poor health outcomes are often exacerbated by experiences of discrimination, stressful socioenvironmental conditions, and lack of healthcare access [6–8]. Yet, the extent of disparities in outcomes and access for SGM populations is unknown due to the lack of sexual orientation and gender identity (SOGI) collection in census information, in Surveillance, Epidemiology, and End Results Programs [9], and in clinical research data.

Systematic collection of SOGI data in clinical research trials and patient assistance programs offers numerous potential benefits, including the assurance that the SGM community is included in healthcare/research settings as well as gaining a better understanding of how to improve on health inequalities. SOGI data increase awareness of diversity in patient inclusion in healthcare settings, how patients are treated, and their outcomes. Data sharing can allow for stronger patient-provider relationships and for appropriate targeted care or preventative services in a representative and inclusive environment [10]. If these data are not collected, professionals in healthcare and research settings are unable to measure who has access to their practices, which patients can gain access through patient assistance programs, or the generalizability of findings to SGM patient communities. Importantly, a request for optional reporting of SOGI data represents a form of inclusion: to be measured is to be counted, and to be counted is to be included.

Despite recommendations by the National Institutes of Health (NIH) [11], National Academies of Sciences, Engineering, and Medicine [12,13], National Science and Technology Council [14], and Society of General Internal Medicine [15], patient SOGI data are not currently being collected in most clinical research or patient assistance settings [16,17]. The overall aim of this real-world, US-based, observational study was to assess the feasibility and outcomes of SOGI data collection in 2 distinct environments: (1) 2 clinical research study programs and (2) a nonprofit patient assistance organization.

## Materials and methods

### Site participation

This survey study involved 2 separate, distinct surveys (1 clinical research survey and 1 patient assistance program survey) and 3 sources of participant responses. No individual participated in both surveys. The 2 participating clinical studies were a phase 2a cough study (sponsored by Genentech, NCT05660850) and a phase 1 cardiac study (sponsored by Genentech, ISRCTN10520571). The patient assistance program web survey was conducted by the Genentech Patient Foundation, an independent nonprofit 501(c)(3) organization that provides free Genentech medication to individuals who qualify based on insurance and financial criteria. The rationale for including 2 distinct surveys with differing respondent compositions was to examine potential variations in the acceptability of SOGI-related questions across diverse populations and settings.

### Clinical research study survey, data collection, and participant eligibility

The clinical research study survey was developed by an internal working group with questions and terminology based on insights from clinical research site interviews and internal groups as well as information from the NIH PhenX Toolkit [18] and Clinical Data Interchange Standards Consortium global data standards (S1 File). Literature reviews and discussions with clinical research centers and organizations were conducted to address current practices and gaps in SOGI data collection. Survey questions provided to participants for the clinical research study pilot programs are in the S2 File.

Data were collected on US patients aged ≥18 years from December 2022 to December 2023. In addition to providing basic demographic data (i.e., age, sex, race), SOGI collection was optional and information was self-reported, mirroring the collection of data on race and ethnicity. Language on the collecting and recording of demographic, race and ethnicity, gender identity, and sexual orientation information was added to clinical site protocols. Sites, institutional review boards, or patients could opt out of data collection if local policy did not allow for collection, if patients did not feel safe disclosing information in their local area, or for other undisclosed reasons. Privacy safeguards were similar to those for other sensitive information (e.g., race, ancestry), as outlined by the General Data Protection Regulation.

Participants at clinical research sites were provided one-time, drop-down survey questions in the presence of research study staff who could explain survey terms or answer questions. Participants chose 1 response to each SOGI-related question.

### Patient assistance program survey, data collection, and participant eligibility

The Genentech Patient Foundation patient assistance program survey was developed using input from focus groups including patients, care partners, internal experts, and the general public using questions based on the race and ethnicity sections of the 2020 US census. Survey questions are in S3 File.

The patient assistance program survey was a web-based, one-time survey completed without the presence of a healthcare provider by participants receiving medications from the program. Any person in the US or US territories who applied to the patient assistance program using the online enrollment form could participate in the survey. Data were collected from January 2023 to December 2023. SOGI collection was optional and self-reported, mirroring the collection of race and ethnicity data; additional demographics, such as age, were not included as part of the survey. Privacy safeguards were similar to those for other sensitive information as outlined by the General Data Protection Regulation.

### Ethical review

Clinical studies were approved by institutional review boards (phase 2a: Advarra; phase 1: WCG) and conducted in accordance with the principles of the Declaration of Helsinki as revised in 2013. Administrative, physical, and technical safeguards were upheld to protect participant confidentiality. All survey participants provided written informed consent, which was obtained through an institutional review board–approved consent form at the clinical site and witnessed by the clinical staff. Clinical research sites included the recording of age, sex, and optional self-reported SOGI information in the informed consent form. Participants received no stipend. S4 File provides the protocol and informed consent language on optional SOGI data collection. There were no questions from the US Food and Drug Administration or institutional review board regarding SOGI data collection.

Participants in the patient assistance program survey were presented with consent language prior to the survey. The recording of race, ethnicity, sexual orientation, and gender identity data was included in the informed consent. Participants were notified that their involvement in the survey would not affect access to the foundation and were also provided a link to Genentech’s privacy policy. Once a participant provided electronic consent, they were allowed access to the survey.

### Statistical analysis

Survey responses from the 2 clinical research study sites were collected in isolation from one another. Participant responses from the patient assistance program were aggregated nationally. Individual survey items were analyzed using descriptive statistics. Frequency counts and percentages were described for ordinal and categorical variables. All analyses were conducted from November 2023 to March 2024.

## Results

### Clinical study surveys

A total of 33 participants completed the clinical research study surveys: 17 from the cough study and 16 from the cardiac study. All participants answered every question, indicating strong overall engagement.

**Fig 1:**
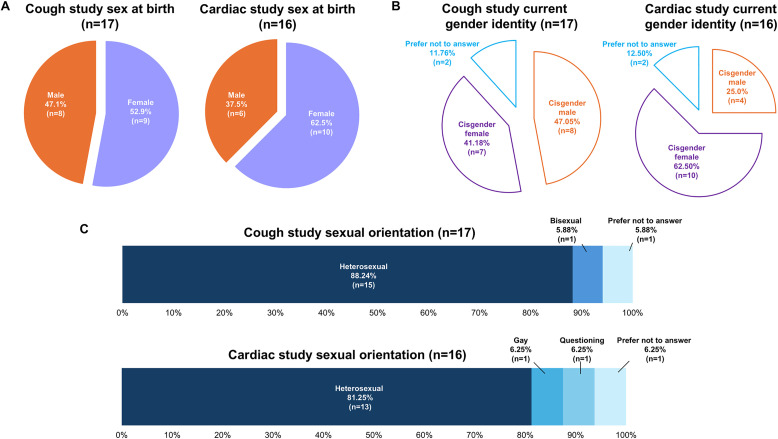
Clinical research survey responses to (A) sex at birth, (B) gender identify, and (C) sexual orientation.

A total of 57.58% of participants in both clinical studies reported their sex at birth as female (Fig 1A). All participants directly answered gender identity questions (Fig 1B); a total of 15 (88.23%) and 14 participants (87.50%) in the cough and cardiac studies, respectively, identified as cisgender. Two participants in each survey chose “Prefer not to answer” in the gender identity section. Among the clinical research survey participants, all participants directly answered questions on sexual orientation (Fig 1C). In the cough study, 15 participants (88.24%) identified as heterosexual and 1 (5.88%) as bisexual; 1 participant (5.88%) chose “Prefer not to answer.” In the cardiac study, 13 participants (81.25%) identified as heterosexual, 1 (6.25%) identified as gay, 1 (6.25%) identified as questioning, and 1 (6.25%) chose “Prefer not to answer.”

Racial and ethnic profiles of the clinical research survey participants are detailed in Table 1. A total of 27 participants (81.82%) were White, 5 (15.15%) were Black, and 1 (3.03%) did not report their race. Combined, 29 participants (87.88%) were not Hispanic or Latino, 3 (9.09%) were Hispanic or Latino, and 1 (3.03%) did not report their ethnicity. A total of 47.06% of participants in the cough study and 56.25% in the cardiac study were 65–74 years old.

**Table 1:** Participant demographics for clinical research survey.

Demographics, no. (%)	Cough study (n = 17)	Cardiac study (n = 16)	Total (n = 33)
Sex			
Male	8 (47.05)	6 (37.50)	14 (42.42)
Female	9 (52.94)	10 (62.50)	19 (57.58)
Age			
84−75 years	3 (17.65)	0	3 (9.09)
74−65 years	8 (47.06)	9 (56.25)	17 (51.52)
64−55 years	5 (29.41)	5 (31.25)	10 (30.30)
54−45 years	1 (5.88)	1 (6.25)	2 (6.06)
44−35 years	0	0	0
34−25 years	0	1 (6.25)	1 (3.03)
Ethnicity			
Hispanic or Latino	0	3 (18.75)	3 (9.09)
Not Hispanic or Latino	16 (94.12)	13 (81.25)	29 (87.88)
Not reported	1 (5.88)	0	1 (3.03)
Race			
White	11 (64.71)	16 (100)	27 (81.82)
Black	5 (29.41)	0	5 (15.15)
Not reported	1 (5.88)	0	1 (3.03)

### Patient assistance program survey

**Fig 2:**
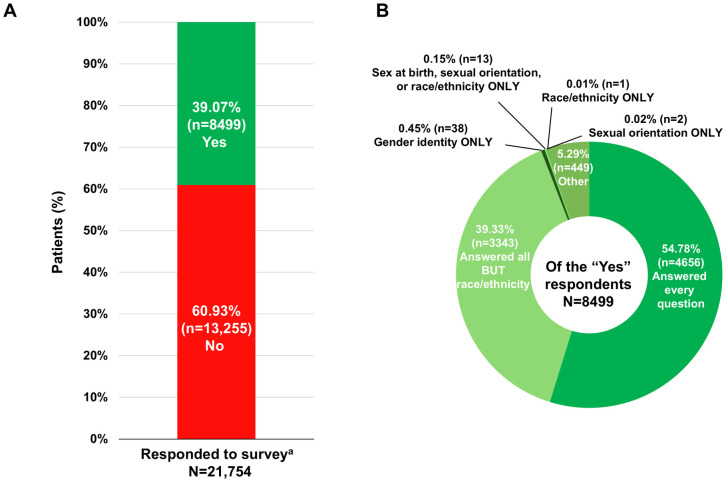
Patient assistance program survey response rates. ^a^ Patients were included as responders if they answered at least 1 question in the survey.

Of the 21,754 individuals who were presented with the survey, 8499 (39.07%) responded to ≥1 question (i.e., participated in the survey) (Fig 2). Of the 8499, the majority (4656 [54.78%]) answered every question; 3343 participants (39.33%) chose to answer sex at birth, gender identity, and sexual orientation questions but did not provide race or ethnicity information. A small proportion of participants only answered 1 section of the survey: 10 (0.12%) only answered what their sex was at birth questions, 38 (0.45%) only answered gender identity questions, 2 (0.02%) only answered sexual orientation questions, and 1 (0.01%) only answered race and ethnicity questions. The response rates suggest robust engagement across survey sections.

**Fig 3:**
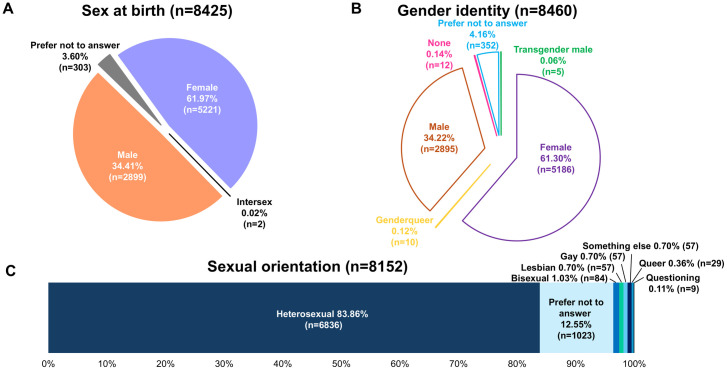
Patient assistance program survey responses to (A) sex at birth, (B) gender identity, and (C) sexual orientation.

A total of 5221 (61.97%) participants reported their sex at birth as female, 2899 (34.41%) male, 303 (3.60%) chose “Prefer not to answer” (Fig 3A), and 2 (0.02%) intersex. A total of 8460 participants (99.54%) responded to gender identity questions (Fig 3B); of these respondents, 8108 (95.86%) chose an answer other than “Prefer not to answer.” The majority of participants self-identified their gender as female (61.30%) or male (34.22%); 10 participants (0.12%) identified as genderqueer and 5 (0.06%) as transgender male. Twelve participants (0.14%) did not feel that any of the options described their gender identity, and 352 (4.14%) chose “Prefer not to answer.”

Of the 8499 survey respondents, 8152 (95.92%) answered sexual orientation questions (Fig 3C); of these respondents, 7129 (87.45%) chose an answer other than “Prefer not to answer.” The majority of participants identified as heterosexual (80.43%), and 57 participants (0.70%) identified as gay, lesbian, or something else. A total of 84 participants (1.03%) identified as bisexual, 29 (0.36%) as queer, and 9 (0.11%) as questioning; 1023 participants (12.55%) chose “Prefer not to answer.”

Racial and ethnic profiles of the patient assistance program survey participants are detailed in Table 2. A total of 825 participants (17.25%) identified as being of Hispanic/Latinx/Spanish origin. Most participants identified as White (59.92%), 18.03% were Black/African American, 4.40% were Asian, 1.42% were Native American/Alaska Native, 0.29% were Native Hawaiian/Pacific Islander, and 2.37% identified as other. A total of 437 participants (10.69%) chose “Prefer not to answer.” The 117 participants (2.86%) who identified as multiracial are further categorized in Table 2.

**Table 2:** Patient assistance program survey participant ethnicity and race demographics.

Demographic, no. (%)	Participants
Ethnicity	n = 4939
Hispanic/Latinx/Spanish	825 (17.25)
Not Hispanic/Latinx/Spanish	4087 (82.75)
Race	n = 4087
White	2449 (59.92)
Black/African American	737 (18.03)
Asian	180 (4.40)
Other	97 (2.37)
Native American/Alaska Native	58 (1.42)
Native Hawaiian/Pacific Islander	12 (0.29)
Prefer not to answer	437 (10.69)
Multiracial	117 (2.86)
White; Other	34 (29.06)
White; American Indian/Alaska Native	27 (23.08)
White; Black/African American	19 (16.24)
White; Asian	8 (6.84)
White; Black/African American/American Indian/Alaska Native	5 (4.27)
Black/African American; Other	4 (3.42)
Black/African American; Asian	3 (2.56)
American Indian/Alaska Native; Other	3 (2.56)
White; Black/African American; Other	2 (1.71)
White; American Indian/Alaska Native; Other	2 (1.71)
Black/African American; American Indian/Alaska Native	2 (1.71)
Asian; Other	2 (1.71)
White; Other; Prefer not to answer	1 (0.85)
White; Black/African American; Prefer not to answer	1 (0.85)
White; Black/African American; Asian; Other; Prefer not to answer	1 (0.85)
Native Hawaiian/Pacific Islander; Other	1 (0.85)
Asian; Native Hawaiian/Pacific Islander	1 (0.85)
American Indian/Alaska Native; Native Hawaiian/Pacific Islander	1 (0.85)

## Discussion

To our knowledge, this study is among the first to look at participant response rates to questions on SOGI in both clinical research study and patient assistance program settings. A high proportion of participants were willing to disclose information on gender identity and sexual orientation in addition to race and ethnicity.

An interesting observation from our clinical research survey was that >60% of respondents were born before 1970. While older generations have a higher nonresponse rate to SOGI questions [19,20], the clinical research survey results suggest that more people aged >50 years are willing to disclose this information in the presence of a healthcare provider. Given the small sample size of the clinical research survey (n = 33), future studies with larger participant populations may offer greater insight into the potential influence of age in disclosing SOGI information. As data on participant age were not collected in the patient assistance program survey, more research on responsiveness between age brackets is needed to fully understand the willingness of participants to respond to these surveys.

An important goal of including SOGI questions in clinical research– and patient assistance–based surveys is to understand key health equity and disparity issues across the population. According to the Pew Research Center survey, approximately 7% of the US population self-identifies as lesbian, gay, or bisexual [21], and of those individuals born between 1997 and 2012, > 1 in 5 consider themselves to be lesbian, gay, bisexual, transgender, queer, or some other sexual orientation besides heterosexual [22]. Including participants with different SOGIs may help researchers understand the response to treatments and interventions for populations with different social drivers of health.

Comprehensive and population-level information about the incidences and outcomes of health conditions across patients with different gender identities and sexual orientations is limited due to the lack of SOGI data collection. For example, in oncology, the healthcare and research field must rely on relatively small studies on disparities in the incidence of cancer and outcomes of treatment in SGM people, which can lead to varied results. Evidence on the incidence or risk of breast cancer in transgender individuals compared with cisgender women is mixed, and it is not clear how or whether hormone therapy used in gender-affirming care contributes to the likelihood of developing cancer [23–26]. This ambiguity highlights the need for SOGI data collection to accurately estimate disease burden and outcomes in patients who may most benefit from treatment.

The ability of healthcare providers and patient assistance programs to address SGM patients’ needs and improve access to health care starts with creating an environment that is representative and inclusive of the SGM community. Studies have shown that people who are SGM are often underserved and that these populations might experience discrimination or microaggressions when seeking services from institutions or healthcare-related foundations [27,28]. As a result, SGM patients often use environmental cues to determine comfortability and acceptance, including questionnaires on SOGI [29,30]. A key takeaway from this study is that individuals are willing to self-identify as transgender. This is important as the difficulty of assessing representative and inclusive healthcare is especially prevalent among transgender individuals.

When surveyed, 19% of transgender people reported refusal of care, 28% reported harassment, and 50% reported having to teach their medical providers about care of transgender people while also facing harassment and discrimination [31]. Providing a space (e.g., demographics survey) for patients to voluntarily disclose SOGI-related information takes the onus off the patient to provide this information and works to ensure that all patients can receive the same level of compassionate and empathetic care. An important goal is for SGM individuals to feel safer and confident in fully using tools for healthcare access, including but not limited to patient assistance programs that can provide access for those with financial barriers. The inclusion of SOGI questions and allowing patients to voluntarily disclose their SOGI information may create a safer, more comfortable environment, and the identity knowledge gained by the provider will aid in giving patients the most appropriate care, which may ultimately lead to better health outcomes.

Currently, the majority of clinical trials and healthcare research do not account for distinctions between sex and gender [32]. In a study that evaluated over 128 major oncology clinical trials that led to US Food and Drug Administration approval of treatments, none of the trials described the collection of sex and gender data, distinguished between sex and gender, or reported any information on the gender of participants, and over one-third had at least 1 inconsistency in the use of sex and gender terms [16]. These gaps are present in other therapy areas, including but not limited to COVID-19 [33], cardiovascular disease [15,34,35], aging/Alzheimer disease [36], and autoimmune disease [37].

Additionally, many providers and researchers are hesitant to implement SOGI questions in their practice. Common concerns include offending patients or leaving patients susceptible to discrimination [38,39]. Despite the unease of providers, only a small minority of patients who were previously presented with SOGI questions reported feeling distressed, and the majority understood the importance of data collection and were willing to participate [40–42].

Over 95% of participants in either survey answered questions on sexual orientation or gender identity, demonstrating proof of concept that participants are willing to answer SOGI questions in a variety of settings. This is consistent with previously reported data from clinical and real-world studies suggesting that people are comfortable disclosing their SOGI status [40–45].

The preliminary feasibility of collecting SOGI data in a clinical research setting has led to the subsequent addition of optional SOGI data collection language in Genentech’s research and early development protocol template and informed consent forms (S4 File). Efforts to expand into other protocol templates are underway. In order to expand into global studies, more work is needed to understand privacy, legal, and regulatory concerns and translations of SOGI terminology for countries outside of the US and languages other than English.

Although the clinical research sample size was 33 patients, the clinical research and patient assistance program surveys had an interesting difference in racial and ethnic diversity. The intersectionality of race and ethnicity and SOGI is an important facet of all aspects of the healthcare ecosystem; male and female sexual minorities of color have a higher prevalence of negative health behaviors, outcomes, and healthcare access and utilization [4]. The clinical research studies primarily included participants who were White and not Hispanic or Latino (consistent with other reports on the lack of representation in clinical trials [46,47]), whereas participant makeup in the patient assistance program survey was similar to US Census Bureau reports on race and Hispanic origin [48].

While previous studies have suggested that race and ethnicity are related to nonresponse rates for SOGI questions [49], both surveys recorded high response rates for SOGI-related questions. The results of our surveys suggest that individuals are willing to report their sexual and gender identities, regardless of race or ethnicity, and are consistent with results in previous publications [41]. One limitation of our study is that we did not correlate SOGI responses to diversity and do not know whether those who identified as sexual minorities also identified as racial or ethnic minorities. Continued research on the relationship between SOGI and racial information disclosure is warranted.

An interesting observation from the patient assistance program survey is that more participants were willing to disclose SOGI information than race and ethnicity. While federal regulations and the NIH require clinical research to report the race and ethnicity of participants [50–53], this requirement does not extend to patient assistance programs. The higher response rate for SOGI vs race and ethnicity questions observed in this study was unexpected, given the voluntary nature of the survey and the prevalence of race and ethnicity disclosure in the healthcare field, and may have been impacted by user experience of the digital survey. Additionally, the reporting of sex and/or gender is often a requirement on many intake forms, including primary care clinics, insurance enrollment, government and public services, or education institutions. The perceived ubiquity of these types of questions may have influenced large numbers of respondents to voluntarily answer gender identity questions. These results support the importance of giving people the opportunity to self-identify and their willingness to do so in different settings.

In addition to healthcare benefits, the collection of optional self-reported SOGI data is ethically important. Such methods respect the autonomy of patients by allowing them to make the final decision on what information they choose to share and empower them to speak about their identity. Equal access to health care is a form of distributive justice. All individuals should have access to the same level of healthcare services and resources, regardless of their identity. Collecting SOGI data may protect individuals from discrimination through inclusion.

### Limitations

Our study has several limitations. The clinical research surveys were conducted in small sample sizes; a current goal is to continue adding SOGI questions to more clinical research studies to not only enhance the dataset but also explore different trials outside of cardiac and cough studies. All surveys were only conducted in the United States and may not reflect global feasibility or results.

The clinical research study surveys and patient assistance program surveys were conducted with 2 unique user experiences, which may have impacted the understanding and willingness of the information participants chose to disclose. The clinical research survey was provided in person in a clinical setting; however, it cannot be assumed that patients asked research staff for help understanding information (e.g., the use of cisgender) while completing the survey. The use of wording in the surveys is important; terms such as intersex, cisgender, or transgender may not be known to the survey participants and may have affected a participant’s decision to provide information. As additional data are obtained, the SOGI questions will evolve to ensure they are understandable, inclusive, and accessible to as many people as possible.

The patient assistance program survey was only presented to respondents who digitally enrolled in the program, omitting patients who enrolled using fax or mail and may have limited access to digital tools. This may have introduced sampling bias due to not including patients without technology access. Age demographics were not collected in the patient assistance program survey but may be a consideration for subsequent initiatives in order to provide readers with greater contextual representation of survey participants.

Differences in demographic factors, settings, and survey composition may have influenced respondents’ willingness to participate and/or the nature of their responses. Recognizing these influences is important for informing future survey designs and ensuring that SOGI data collection efforts are both inclusive and appropriately tailored to diverse respondent groups.

Both surveys included grouped identity categories such as “Prefer not to answer,” “something else,” or “none of the above.” While this approach can support data simplification or protect respondent anonymity, it may obscure meaningful distinctions among SGM participants not adequately captured by binary or predefined options. Future survey designs should explore more nuanced and inclusive approaches to identity categorization.

Intersecting identities, such as age, race, socioeconomic status, and disability, can play a large role in shaping respondents’ comfort and feasibility when disclosing SOGI-related information, and these factors may influence individuals’ willingness to participate. Recognizing these dynamics is vital for interpreting response patterns and ensuring inclusive data collection. This is important for both clinical trials, which are regulated by their inclusion/exclusion criteria, and in real-world populations, which can provide better insights on which populations are the most comfortable disclosing SOGI data. Future research should prioritize intersectional approaches that explore how overlapping identities shape experience with SOGI data collection, including engaging diverse communities in survey design and developing adaptive methodologies.

## Conclusions

These data reflect that patients in clinical research and patient assistance program settings are willing to self-disclose SOGI information. Adding SOGI questions to demographic forms can help discover gaps in access and increase awareness of medical needs to help guide research campaigns and equitable access to patient assistance programs and clinical research studies. To build on this foundational survey, further adoption is needed by clinical research, patient assistance programs, and throughout the healthcare ecosystem.

## Supporting information

S1 File Preferred terminology for survey questions. (DOCX)

S2 File Optional SOGI questionnaire for clinical research survey. (DOCX)

S3 File Patient assistance program survey. (DOCX)

S4 File Optional SOGI data collection language added to Genentech research and early development template protocol and informed consent forms. (DOCX)
