# Supplementary material for: Sexual orientation and gender identity data: An observational study assessing the feasibility of SOGI collection in clinical research and patient assistance programs
Source: PLoS One. 2025 Oct 22;20(10):e0332805. doi: 10.1371/journal.pone.0332805 (PMC12543137; doi:10.1371/journal.pone.0332805)
Supplement: S1 File — (DOCX) [file pone.0332805.s001.docx]

**S1 File. Preferred terminology for survey questions**

| **Term** | **Definition^a^** |
| --- | --- |
| Bisexual | A person who has the potential to be physically, romantically, and/or emotionally attracted to people of more than one gender, not necessarily at the same time, in the same way, or to the same degree |
| Cisgender | An adjective used to describe people who are not transgender. A cisgender person is a person whose gender identity is aligned with the sex they were assigned at birth |
| Gay | An adjective used to describe a person whose enduring physical, romantic, and/or emotional attractions are to people of the same sex |
| Gender expression | External manifestations of gender, expressed through a person’s name, pronouns, clothing, haircut, voice, and/or behavior |
| Gender identity | A person’s internal, deeply held knowledge of their own gender |
| Genderqueer^b^ | Of, relating to, or being a person whose gender identity may not be categorized in binary terms |
| Heterosexual | An adjective used to describe a person whose enduring physical, romantic, and/or emotional attraction is to people of a sex different than their own |
| Intersex | An adjective used to describe a person with one or more innate sex characteristics, including genitals, internal reproductive organs, and chromosomes, that fall outside of traditional conceptions of male or female bodies |
| Lesbian | A woman whose enduring physical, romantic, and/or emotional attraction is to other women |
| Queer | An adjective used by some people, particularly younger people, whose sexual orientation is not exclusively heterosexual |
| Questioning | Relating to individuals in the process of exploring their sexual orientation and/or gender identity |
| Sex | Infants are assigned a sex at birth, “male” or “female,” based on the appearance of their external anatomy, and an M or an F is written on the birth certificate. However, the development of the human body is a complex process, and sex is not solely determined by anatomy, nor is it strictly binary |
| Sexual orientation | The scientifically accurate term for a person’s enduring physical, romantic, and/or emotional attraction to another person |
| Transgender/trans | Of, relating to, or being a person whose gender identity differs from the sex they were assigned at birth |
| Transgender man | A person who identifies as a man and was assigned female sex at birth |
| Transgender woman | A person who identifies as a woman and was assigned male sex at birth |

^a^ Definitions are from the Gay & Lesbian Alliance Against Defamation organization and website.

^b^ Definition is from the Merriam Webster dictionary and website.
