# Supplementary material for: Sexual orientation and gender identity data: An observational study assessing the feasibility of SOGI collection in clinical research and patient assistance programs
Source: PLoS One. 2025 Oct 22;20(10):e0332805. doi: 10.1371/journal.pone.0332805 (PMC12543137; doi:10.1371/journal.pone.0332805)
Supplement: S2 File — (DOCX) [file pone.0332805.s002.docx]

**S2 File. Optional SOGI questionnaire for clinical research survey**

1. What is your sexual orientation?
   1. Heterosexual
   2. Lesbian
   3. Gay
   4. Bisexual
   5. Queer
   6. Questioning
   7. Other, please specify
   8. Prefer not to answer
2. What is your current gender identity?
   1. Cisgender male
   2. Cisgender female
   3. Transgender male
   4. Transgender female
   5. Nonbinary
   6. Other, please specify
   7. Prefer not to answer
