# Supplementary material for: Sexual orientation and gender identity data: An observational study assessing the feasibility of SOGI collection in clinical research and patient assistance programs
Source: PLoS One. 2025 Oct 22;20(10):e0332805. doi: 10.1371/journal.pone.0332805 (PMC12543137; doi:10.1371/journal.pone.0332805)
Supplement: S3 File — (DOCX) [file pone.0332805.s003.docx]

**S3 File. Patient assistance program survey**

1. What is your race/ethnicity? (select all that apply)
   1. Hispanic, Latinx, or Spanish
   2. White
   3. Black or African American
   4. Asian
   5. American Indian or Alaska Native
   6. Pacific Islander
   7. Other
   8. Prefer not to answer
2. What is your sexual orientation? (choose one)
   1. Heterosexual
   2. Lesbian
   3. Gay
   4. Bisexual
   5. Queer
   6. Something else
   7. Questioning
   8. Prefer not to answer
3. What is your biological sex from birth? (choose one)
   1. Male
   2. Female
   3. Intersex
   4. Prefer not to answer
4. What is your current gender identity? (choose one)
   1. Male
   2. Female
   3. Transgender male
   4. Transgender female
   5. Genderqueer
   6. Something else
   7. None of these describe me
   8. Prefer not to answer
