# Supplementary material for: Sexual orientation and gender identity data: An observational study assessing the feasibility of SOGI collection in clinical research and patient assistance programs
Source: PLoS One. 2025 Oct 22;20(10):e0332805. doi: 10.1371/journal.pone.0332805 (PMC12543137; doi:10.1371/journal.pone.0332805)
Supplement: S4 File — (DOCX) [file pone.0332805.s004.docx]

**S4 File.** **Optional SOGI data collection language added to** **Genentech research and early development template protocol and informed consent forms**

Protocol language: Demographic data, including age and sex, will be recorded. If permitted by local Institutional Review Board/Ethics Committee policies, self-reported race and/or ethnicity, gender identity, and sexual orientation will also be recorded for participants willing to provide this information.

Informed consent form language: Recording of age and sex and optional recording of self-reported race and/or ethnicity, gender identity, and sexual orientation.
